# 

**Published:** 1879-12

**Authors:** 


### Table of Contents.

ORIGINAL COMMUNICATIONS.

Laryngitis Catarrhalis—Pseudo Croup. By W. Salm, M.D., Atlanta, Ga. .......... 515

REPORTS OF SOCIETIES.

Atlanta Academy of Medicine .......... 517
St. Louis Medico Chirurgical Society .......... 518
Buffalo Medical Association .......... 526
A Clinical Lecture Delivered at the Bellevue Hospital, New York .......... 530

SELECTIONS

The Treatment of Post-Nasal Catarrh .......... 546
Recent Progress in the Treatment of Mental Diseases .......... 453
Cysticercus Cellulosæ .......... 458

EDITORIAL.

American Public Health Association .......... 460
Meteorological Report .......... 462
Editorial Correspondence .......... 563

BIBLIOGRAPHICAL.

Yellow Fever a Nautical Disease .......... 567
Medical Chemistry .......... 567
The Treatment of Diseases by the Hypodermic Method .......... 567

EXECRPTA.

Quinia and Amyl Nitrite in Pertussis .......... 568
The Immediate as Distinguished from the Permanent Treatment of Disease .......... 569
Case of Insufficiency of the Mitral Valves .......... 570
Dangers from Turpentine Vapor .......... 573
Prescription for Dysmenorrhœa .......... 573
Treatment of Pertussis .......... 574
Ipecac as a Hæmostatic .......... 574
How to Prevent Mammary Abscess .......... 575
Thymoline Soap .......... 575
Remarkable Menstruation .......... 575
Automatic Medical Electricity .......... 576
Cellulitis of the Neck .......... 577
Gynecologists .......... 578
Citron Juice .......... 578
A Milk Test .......... 578
Russian Gargle .......... 578
Salisylic Acid as an Anti-Scorbutic .......... 578

